# Functional Walking Capacity as a Screening Indicator for Peripheral Arterial Disease in Patients with Type 2 Diabetes Mellitus: Development of a Clinical Risk Equation

**DOI:** 10.3390/diagnostics16121750

**Published:** 2026-06-06

**Authors:** Busaba Chuatrakoon, Sothida Nantakool, Nipawan Waisayanand, Termpong Reanpang, Lalisa Wanichsuksombat, Supatcha Konghakote, Khun Phisitbuntoon, Nichakorn Jaiin, Surangsiri Rattanaphan

**Affiliations:** 1 Integrated Neuro-Musculoskeletal, Chronic Disease, and Aging Research Engagement Center (ICARE Center), Department of Physical Therapy, Faculty of Associated Medical Sciences, Chiang Mai University, Chiang Mai 50200, Thailand; busaba.c@cmu.ac.th (B.C.); lalisa_wanich@cmu.ac.th (L.W.); supatcha.k@cmu.ac.th (S.K.); khun_phisit@cmu.ac.th (K.P.); nichakorn_jaiin@cmu.ac.th (N.J.); surangsiri_r@cmu.ac.th (S.R.); 2 Division of Endocrinology, Department of Internal Medicine, Faculty of Medicine, Chiang Mai University, Chiang Mai 50200, Thailand; nipawan.w@cmu.ac.th; 3 Division of Vascular and Endovascular Surgery, Department of Surgery, Faculty of Medicine, Chiang Mai University, Chiang Mai 50200, Thailand; termpong.reanpang@cmu.ac.th

**Keywords:** peripheral arterial disease, type 2 diabetes mellitus, functional walking capacity, incremental shuttle walk test, risk stratification

## Abstract

**Background/Objectives:** Peripheral arterial disease (PAD) is a common complication in patients with type 2 diabetes mellitus (T2DM) and is associated with substantial morbidity. Standard diagnostic tools such as the ankle–brachial index (ABI) may be limited in certain clinical settings. Therefore, simple and practical approaches for identifying individuals at increased risk of PAD are needed. This study aimed to examine the association between functional walking capacity and PAD and to develop a simple clinical risk equation for identifying PAD risk in patients with T2DM. **Methods:** A cross-sectional analytical study was conducted in 223 patients with T2DM attending an outpatient clinic. PAD was diagnosed using ABI and toe–brachial index measurements. Functional walking capacity was assessed using the Incremental Shuttle Walk Test (ISWT). Multivariable logistic regression analysis was used to identify factors associated with PAD and to develop a risk equation. Model performance was evaluated using receiver operating characteristic (ROC) curve analysis. **Results:** An ISWT distance ≤ 215 m was independently associated with PAD (OR = 1.96, 95% CI 1.10–3.50, *p* = 0.022). The developed model demonstrated fair discriminative ability (AUC = 0.67). At a probability cutoff of 0.57, the model showed a sensitivity of 53.8% and a specificity of 76.4%. **Conclusions:** Reduced functional walking capacity was associated with PAD in patients with T2DM. The proposed equation demonstrated fair discriminatory ability and may provide preliminary information for PAD risk identification; however, further validation is required before clinical implementation.

## 1. Introduction

Peripheral arterial disease (PAD) is a common manifestation of systemic atherosclerosis and represents a major public health concern due to its association with increased risks of cardiovascular morbidity, mortality, and functional decline [1,2]. The global prevalence of PAD has increased substantially, affecting more than 200 million people worldwide, particularly among older adults and individuals with cardiometabolic risk factors [3,4]. Patients with type 2 diabetes mellitus (T2DM) are at particularly high risk due to chronic hyperglycemia, endothelial dysfunction, and accelerated atherosclerotic processes, which contribute to disease progression and adverse outcomes such as foot ulcers, lower-limb amputation, and premature mortality [5,6]. Both T2DM and PAD are characterized by chronic low-grade inflammation and vascular dysfunction, which contribute to disease progression and functional impairment.

Early identification of PAD is essential for preventing disease progression and improving clinical outcomes. The ankle–brachial index (ABI) and toe–brachial index (TBI) are widely used for PAD detection [7,8]; however, their application may be limited in certain clinical contexts [9,10]. In patients with diabetes, arterial calcification may lead to falsely elevated ABI values, reducing diagnostic accuracy [5]. Consequently, some individuals with PAD may remain undetected despite ABI screening, particularly in the presence of medial arterial calcification. In addition, the requirement for specialized equipment and trained personnel may restrict their use in primary care or resource-limited settings. Taken together, these limitations highlight the potential value of complementary approaches that capture the functional consequences of vascular disease and help identify individuals at increased risk of PAD who may benefit from further vascular assessment.

Functional walking capacity reflects the integrated performance of cardiovascular, pulmonary, and musculoskeletal systems and has been widely used as an indicator of overall physical function. In individuals with PAD, reduced blood flow to the lower extremities can impair oxygen delivery during physical activity, resulting in diminished exercise tolerance and walking performance [11,12]. Field-based assessments, such as the six-minute walk test (6MWT) and the incremental shuttle walk test (ISWT), are practical tools for evaluating functional capacity in clinical and community settings [13,14].

The progressive nature of the ISWT, characterized by gradually increasing walking demands, may make it particularly suitable for identifying functional limitations associated with impaired lower-extremity perfusion. Emerging evidence suggests that ISWT may be more sensitive than 6MWT in detecting functional impairment in individuals with PAD, particularly in those with asymptomatic disease. A previous study demonstrated that individuals with asymptomatic PAD exhibited significantly reduced walking performance when assessed using ISWT, while no significant difference was observed with 6MWT, indicating that ISWT may better reflect early functional limitations [15]. In clinical practice, the identification of individuals at increased risk of PAD remains challenging, particularly in the early stages of the disease when symptoms may be absent or nonspecific. Many patients with T2DM do not present with classic intermittent claudication, leading to delayed diagnosis and under-recognition of the condition. Moreover, reliance on vascular diagnostic tools alone may not fully capture the functional impact of the disease, especially in settings where routine screening is not systematically implemented. As a result, there is increasing interest in incorporating functional assessments into risk identification strategies.

Functional performance measures have the potential to reflect the integrated effects of cardiovascular, metabolic, and musculoskeletal impairments associated with PAD. Unlike isolated physiological measurements, functional tests may provide a more holistic representation of disease burden and its impact on daily activities. Therefore, integrating functional walking capacity into screening approaches may enhance early detection and support timely referral for confirmatory vascular assessment, particularly in high-risk populations such as individuals with T2DM.

Despite these insights, most existing studies have focused on comparing functional performance between individuals with and without PAD, rather than integrating functional measures into clinically applicable risk identification models. The potential role of functional walking capacity as a screening-oriented indicator for PAD, particularly in high-risk populations such as individuals with T2DM, remains underexplored.

Therefore, this study aimed to examine the association between functional walking capacity and PAD and to develop a simple clinical risk equation incorporating functional performance to identify individuals at increased risk of PAD in patients with type 2 diabetes mellitus.

## 2. Materials and Methods

### 2.1. Study Design and Setting

This study employed a cross-sectional analytical design to investigate the association between functional walking capacity and peripheral arterial disease (PAD) in patients with type 2 diabetes mellitus (T2DM). The study was conducted at the Endocrinology Outpatient Clinic of Maharaj Nakorn Chiang Mai Hospital, Chiang Mai, Thailand.

The study protocol was approved by the Institutional Review Board of the Faculty of Medicine, Chiang Mai University. All participants provided written informed consent prior to participation in the study (NONE-2568-0310).

### 2.2. Sample Size Calculation

The required sample size was estimated based on the anticipated association between reduced functional walking capacity and peripheral arterial disease (PAD), derived from preliminary data (odds ratio ≈ 2.33). Using G*Power software (version 3.1.9.7; Heinrich Heine University, Düsseldorf, Germany) for logistic regression analysis (two-tailed α = 0.05, statistical power = 0.80), a minimum sample size of 223 participants was determined.

Given the number of PAD cases observed in the dataset, the events-per-variable (EPV) ratio was evaluated to assess model stability. As the EPV was relatively limited, the developed model should be interpreted as exploratory and screening-oriented rather than a definitive predictive model.

### 2.3. Participants

A total of 223 patients with T2DM were recruited using a consecutive sampling method. Participants were eligible if they were diagnosed with T2DM according to the American Diabetes Association criteria [5] and were able to walk independently and perform the Incremental Shuttle Walk Test (ISWT).

Exclusion criteria included conditions that could affect walking performance or interfere with PAD assessment, such as severe musculoskeletal disorders, neurological impairments affecting mobility, acute cardiovascular events, or severe respiratory disease.

### 2.4. Outcome Measures

#### 2.4.1. Peripheral Arterial Disease Assessment

Peripheral arterial disease was assessed using the ankle–brachial index (ABI) and toe–brachial index (TBI) following standard vascular assessment procedures [9,10]. Systolic blood pressure was measured at the brachial artery, ankle, and toe arteries. ABI and TBI values were calculated as the ratio of ankle or toe systolic pressure to brachial systolic pressure. PAD was defined as an ABI < 0.90 and/or TBI < 0.70 according to established guidelines [9,10]. ABI and TBI measurements were obtained using an automated vascular screening device (VaSera VS-1500N, Fukuda Denshi Co., Ltd., Tokyo, Japan).

#### 2.4.2. Functional Walking Capacity

Functional walking capacity was assessed using the Incremental Shuttle Walk Test (ISWT) [13]. The test was conducted according to a standardized protocol using an externally paced audio signal, with standardized verbal encouragement provided throughout. Participants walked back and forth along a 10-m course at increasing speeds dictated by the audio signals. The test was terminated when the participant was unable to maintain the required pace or experienced limiting symptoms. The total walking distance (meters) was recorded.

For clinical interpretation, ISWT performance was categorized using a cut point of ≤215 m, derived from the present dataset, to identify reduced functional walking capacity.

#### 2.4.3. Clinical Variables

Demographic and clinical variables were collected through interviews and medical record review, including age, sex, smoking status, hypertension, hyperlipidemia, family history of cardiovascular disease, and physical activity level. Movement behavior was classified as active or sedentary.

### 2.5. Study Procedure

Eligible participants were recruited during routine outpatient visits. After providing informed consent, demographic and clinical data were collected. Participants then underwent PAD assessments using ABI and TBI measurements, followed by evaluation of functional walking capacity using the ISWT. All measurements were performed by trained assessors following standardized procedures. Data collection was conducted between July 2025 and October 2025.

### 2.6. Statistical Analysis

Descriptive statistics were used to summarize participant characteristics. Continuous variables were presented as mean ± standard deviation, while categorical variables were reported as frequencies and percentages.

Multivariable logistic regression analysis was performed to identify factors associated with PAD and to develop a clinical risk equation. Variables included established PAD risk factors (age, sex, hypertension, hyperlipidemia, smoking status, family history, physical activity level, and movement behavior), which were retained based on their clinical relevance, together with ISWT performance to evaluate its additional value beyond established risk factors in identifying individuals at increased risk of PAD.

The predictive performance of the model was evaluated using receiver operating characteristic (ROC) curve analysis. The area under the curve (AUC) was used to assess discriminative ability. Sensitivity and specificity were calculated based on the optimal probability cutoff. ROC analysis was also used to determine the optimal cutoff-point of ISWD. ISWD was subsequently dichotomized based on this threshold for regression analyses.

All statistical analyses were performed using appropriate statistical software, and statistical significance was set at *p* < 0.05. Statistical analyses were performed using SPSS version 30 (IBM Corp., Armonk, NY, USA).

## 3. Results

**Figure 1:**
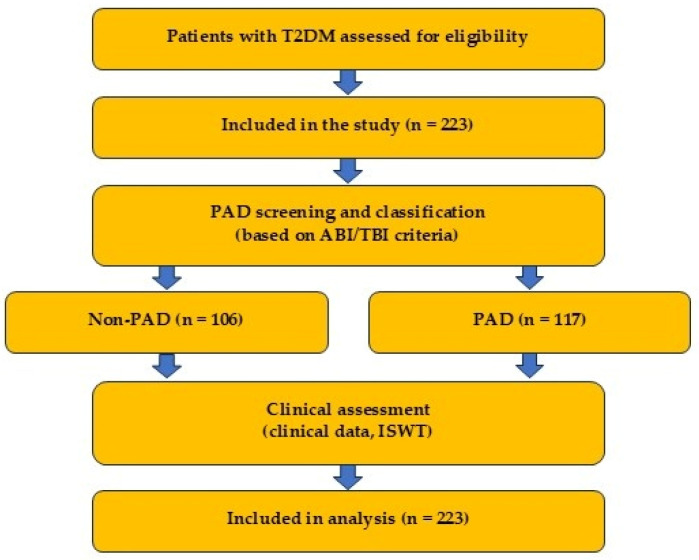
Flow diagram of participant recruitment and classification.

Participants were recruited from the Endocrinology Outpatient Clinic using a consecutive sampling approach and underwent a standardized assessment protocol, including vascular evaluation and measurement of functional walking capacity. The process of recruitment, eligibility confirmation, and classification into peripheral arterial disease (PAD) and non-PAD groups is illustrated in Figure 1. All variables included in the analysis were complete, with no missing data observed.

### 3.1. Participant Characteristics

A total of 223 patients with T2DM were included in this study, comprising 106 participants without PAD and 117 participants with PAD. The demographic and clinical characteristics of the participants are presented in Table 1.

The mean age of the study population was 63.6 ± 8.0 years, with participants with PAD being slightly older than those without PAD (64.6 ± 8.1 vs. 62.8 ± 7.3 years). The proportion of male participants was 38.6% overall, with a higher proportion in the non-PAD group compared to the PAD group (43.4% vs. 34.2%).

Hypertension and dyslipidemia were highly prevalent in both groups. Hypertension was more frequently observed in participants with PAD (73.5%) compared to those without PAD (63.2%), whereas dyslipidemia showed similar distribution between groups. The duration of diabetes mellitus appeared to differ between groups, with a higher proportion of participants with PAD having a disease duration greater than 15 years (47.6% vs. 29.3%). Functional walking capacity, as measured by the incremental shuttle walk distance (ISWD), was lower in participants with PAD compared to those without PAD (222.1 ± 81.4 vs. 250.8 ± 88.9 m).

### 3.2. ROC Analysis and Cutoff-Point Determination for ISWD

The receiver operating characteristic (ROC) analysis demonstrated a modest discriminative ability of ISWD for identifying the outcome of interest, with an area under the curve (AUC) of 0.60 (95% CI: 0.54–0.67). The ROC curve was used to assess the ability of ISWD to distinguish between individuals with and without the condition across a range of threshold values.

The optimal cut-off value, determined based on the Youden index, was 215 m. At this threshold, the sensitivity was 62.6% and the specificity was 58.3%, reflecting the proportions of correctly classified individuals with and without the condition, respectively. This cut-off value was subsequently applied to dichotomize ISWD for inclusion in the multivariable logistic regression analysis (Table 2).

### 3.3. Factors Associated with Peripheral Arterial Disease

Multivariable logistic regression analysis was performed to identify factors associated with PAD (Table 3). Among all variables included in the model, reduced functional walking capacity, defined as ISWD ≤ 215 m, was the only factor significantly associated with PAD.

Participants with ISWD ≤ 215 m had a significantly higher likelihood of PAD compared with those who walked more than 215 m (OR = 1.96, 95% CI 1.10–3.50, *p* = 0.022). Other variables, including age, sex, hypertension, hyperlipidemia, smoking status, family history of cardiovascular disease, physical activity level, and movement behavior, were not statistically significant in the multivariable model.

### 3.4. Prediction Model and Performance

Based on multivariable logistic regression analysis, a clinical risk equation was developed to estimate the probability of PAD. The model incorporated demographic and clinical variables together with functional walking capacity.

(1) Score = −2.446 + 0.0236 (age) − 0.327 (sex) + 1.144 (DM) + 0.605 (HT) − 0.571 (HCL) + 0.072 (smoke1) − 0.335 (smoke2) + 0.307 (family) − 0.410 (act1) − 0.291 (act2) − 0.034 (movement) + 0.675 (ISWT215)

In this model, age was entered as a continuous variable (years). Binary variables were coded as follows: sex (male = 1, female = 0); diabetes mellitus (DM: yes = 1, no = 0); hypertension (HT: yes = 1, no = 0); hyperlipidemia (HCL: yes = 1, no = 0); family history of cardiovascular disease (family: yes = 1, no = 0); smoking status (smoke1: current smoker = 1, otherwise = 0; smoke2: former smoker = 1, otherwise = 0); physical activity level (act1: high physical activity = 1, otherwise = 0; act2: moderate physical activity = 1, otherwise = 0); movement behavior (active = 1, sedentary = 0); and ISWT performance (iswd215: ≤215 m = 1, >215 m = 0).

The predicted probability of PAD can be calculated using the logistic transformation:

(2) Probability = 1/(1 + e^(−Score)^)

Participants with a predicted probability greater than the cutoff value were classified as being at increased risk of PAD.

**Figure 2:**
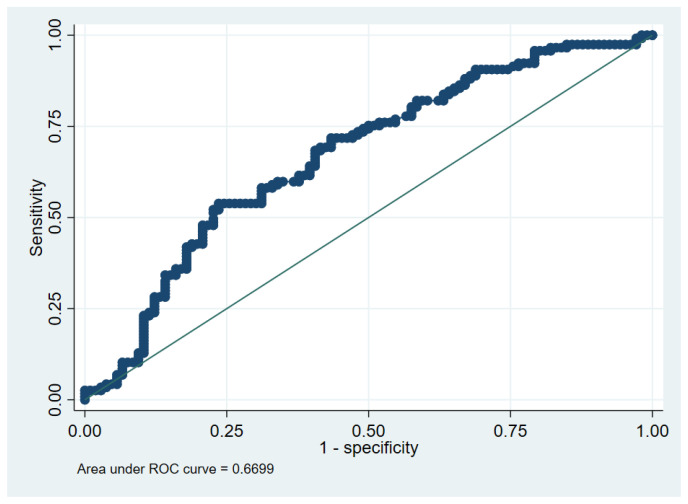
Receiver operating characteristic curve of the risk prediction model for identifying peripheral arterial disease.

The risk prediction model demonstrated a fair discriminative ability for identifying peripheral arterial disease (PAD), as reflected by an area under the receiver operating characteristic (ROC) curve (AUC) of 0.67 (95% CI: 0.60–0.74). The ROC curve analysis was performed to evaluate the model’s ability to distinguish between individuals with and without PAD across a range of threshold values. Based on the Youden index, the optimal cut-off value was identified at 0.57. At this threshold, the model yielded a sensitivity of 53.8% and a specificity of 76.4%, indicating the corresponding proportions of correctly identified PAD cases and non-PAD cases, respectively (Table 4). The ROC curve of the risk prediction model is demonstrated in Figure 2.

## 4. Discussion

The present study demonstrated that reduced functional walking capacity, defined as an ISWT distance ≤ 215 m, was significantly associated with peripheral arterial disease (PAD) in patients with type 2 diabetes mellitus. Individuals with lower walking performance had nearly twofold higher odds of having PAD compared with those with better functional capacity. In addition, the proposed model showed fair discriminatory ability (AUC = 0.67), suggesting its potential utility as an exploratory risk-identification approach for PAD.

A key contribution of this study is the incorporation of functional walking capacity into a clinically applicable risk identification framework. While conventional PAD detection relies primarily on vascular assessments such as the ankle–brachial index (ABI) or toe–brachial index (TBI), these methods may be limited in certain settings, particularly in primary care or resource-constrained environments [9,10]. In contrast, functional assessments such as the Incremental Shuttle Walk Test (ISWT) are simple, low-cost, and require minimal equipment, making them more accessible for routine clinical use [16,17]. The present findings suggest that functional walking performance may provide complementary information beyond traditional vascular measurements and may help identify individuals who warrant further vascular evaluation.

The observed association between reduced walking capacity and PAD can be explained by underlying pathophysiological mechanisms. PAD is characterized by atherosclerotic obstruction of peripheral arteries, leading to reduced blood flow and impaired oxygen delivery to skeletal muscles during physical activity [1,18,19]. This results in early onset of muscle fatigue, decreased exercise tolerance, and reduced walking performance. In individuals with T2DM, these impairments may be further exacerbated by microvascular dysfunction, mitochondrial abnormalities, and structural changes in skeletal muscle, contributing to greater functional limitation [2,20,21]. Consequently, functional walking capacity may reflect the cumulative physiological burden of vascular and metabolic impairment.

The findings of this study are consistent with previous evidence demonstrating that walking performance is closely associated with disease severity and functional limitation in PAD [2,12,22]. In particular, progressive field-based tests such as the ISWT may be more sensitive in detecting early functional impairment compared with self-paced tests, as they impose increasing physiological demands and better reflect cardiopulmonary and musculoskeletal integration [13,14]. This may explain why reduced ISWT performance was significantly associated with PAD in this study, even in the absence of strong associations with traditional risk factors.

Interestingly, conventional cardiovascular risk factors, including age, smoking status, hypertension, and hyperlipidemia, were not statistically significant in the multivariable model. This may be due to the relatively homogeneous risk profile of the study population, as all participants had T2DM and a high prevalence of cardiometabolic comorbidities [23,24]. In such populations, functional measures may capture the integrated effects of disease burden more effectively than individual risk factors alone, thereby providing additional discriminatory value [18].

From a clinical perspective, the proposed model should be considered an exploratory risk-identification approach rather than a validated screening or diagnostic tool. Although the model demonstrated limited sensitivity and moderate specificity, its simplicity and reliance on functional assessment may provide preliminary information for PAD risk identification in settings where vascular diagnostic tools are not readily available. However, further validation is required before clinical application can be considered. Functional walking tests such as the ISWT can be easily implemented in outpatient clinics and community-based programs, potentially facilitating risk identification and referral for further vascular evaluation [17,25]. Nevertheless, the model should be interpreted with caution. The relatively modest AUC indicates only fair discriminatory performance, and the limited events-per-variable ratio suggests that the model is exploratory in nature. Therefore, it should not be used as a standalone diagnostic tool but rather as an exploratory risk-identification approach that warrants further validation.

Beyond its role in screening and risk stratification, the integration of functional walking capacity into clinical assessment may have broader implications for patient management. Functional limitations are closely linked to quality of life, independence, and long-term health outcomes. In patients with T2DM, reduced walking capacity may reflect not only vascular impairment but also the cumulative effects of metabolic dysregulation, physical inactivity, and comorbid conditions [26,27]. Therefore, incorporating functional performance measures into routine clinical evaluation may provide a more comprehensive understanding of patient status and help guide individualized management strategies.

In comparison with traditional screening approaches, the use of functional walking tests offers several advantages. While ABI and TBI provide objective measures of vascular status [9,10], they do not capture the functional consequences of impaired perfusion. In contrast, walking tests assess the integrated response of multiple physiological systems, including cardiovascular, pulmonary, and musculoskeletal components [13,15]. This multidimensional nature may enhance the ability to detect early or subclinical impairment, particularly in populations where vascular measurements may be less reliable. However, it is important to acknowledge that walking performance assessed by the ISWT is not specific to PAD alone. Factors such as cardiorespiratory fitness, diabetic neuropathy, musculoskeletal impairments, motivation, and other comorbid conditions may influence walking performance independently of vascular disease. In the present study, physical activity levels were comparable between participants with and without PAD. Therefore, differences in habitual physical activity, a surrogate marker of cardiorespiratory fitness, are less likely to fully explain the lower ISWT performance observed among participants with PAD. Nevertheless, unmeasured factors may have contributed to the observed differences in walking capacity and should be considered when interpreting the findings. As such, ISWT should be interpreted as a measure of overall functional capacity rather than a PAD-specific indicator and considered within the broader clinical context.

The present findings support further investigation of functional assessment as a potential component of future PAD risk-identification strategies. In clinical settings where access to vascular diagnostic tools is limited, functional walking capacity may provide preliminary information to support PAD risk identification. This approach may be particularly valuable in primary care and community-based settings, where large numbers of patients with T2DM require ongoing monitoring. By prioritizing individuals with reduced functional performance for additional vascular assessment, healthcare providers may inform decisions regarding further vascular assessment and optimize resource allocation [28].

Furthermore, the integration of functional assessment into digital health and telemedicine platforms represents a promising direction for future research and clinical practice. Advances in wearable technology and remote monitoring systems may enable continuous or periodic assessment of walking performance in real-world settings [29,30]. Such approaches could facilitate early detection of functional decline, support personalized intervention strategies, and enhance patient engagement in self-management. Future studies should explore the feasibility and effectiveness of integrating functional walking measures into digital health frameworks for PAD risk assessment.

Finally, although the predictive performance of the proposed model was modest, it is important to consider its intended purpose. The model was designed as a screening-oriented tool rather than a definitive diagnostic instrument. In this context, moderate discriminative ability may still be clinically meaningful, particularly when combined with other sources of information. Further refinement and external validation of the model may improve its performance and support its application in diverse populations. Incorporating additional variables, such as muscle strength, physical performance tests, or biomarkers, may enhance predictive accuracy and should be considered in future research.

### Study Limitations

Several limitations should be considered when interpreting the findings of this study. First, the cross-sectional design precludes causal inference between functional walking capacity and PAD, and the temporal relationship between these variables cannot be established. Second, the study was conducted in a single tertiary care center, which may limit generalizability to other populations, particularly those in primary care or community-based settings. Third, the number of PAD events relative to the number of predictors was limited, which may affect model stability and increase the risk of overfitting. Therefore, the proposed model should be considered exploratory, and its performance may not be generalizable to other populations without further external validation. Fourth, important variables such as muscle strength, physical activity levels, detailed body composition, and specific comorbidities (e.g., diabetic neuropathy and heart failure) were not included in the analysis. These factors may influence walking capacity independently of PAD and could have contributed additional predictive value or residual confounding.

In addition, the ISWT cut-off point was derived from and evaluated within the same study sample. Therefore, the identified threshold should be considered exploratory and may be subject to optimism bias, potentially limiting its external applicability across populations with different demographic and clinical characteristics. Measurement-related factors, including participant motivation and test familiarity, may also have influenced ISWT performance. Finally, although the model demonstrated fair discriminatory ability, internal validation was limited, and external validation in independent cohorts is required before broader clinical implementation can be recommended.

## 5. Conclusions

Reduced functional walking capacity was significantly associated with PAD in patients with T2DM. The proposed model demonstrated fair discriminatory ability and may provide preliminary information for PAD risk identification; however, external validation is required before clinical implementation. Functional walking assessment, particularly the ISWT, may provide preliminary information for PAD risk identification, especially in settings where vascular diagnostic tools are not readily accessible. Nevertheless, the proposed model should be interpreted as an exploratory risk-identification approach rather than a validated screening or diagnostic tool.

Future studies with larger, multicenter, and prospective designs are needed to validate the model and establish its clinical utility. Further refinement incorporating additional clinical and functional variables, as well as integration with digital health technologies, may improve predictive performance and support more personalized risk stratification in patients with T2DM.

## Figures and Tables

**Table 1:** Participant demographics.

Variables	Sub-Variables	Total (*n* = 223)	No PAD (*n* = 106)	PAD (*n* = 117)
Age (y), mean ± SD	-	63.6 ± 8.0	62.8 ± 7.3	64.6 ± 8.1
Sex (male) (*n*, %)	-	86 (38.6%)	46(43.4%)	40 (34.2%)
Underlying diseases (*n*, %)	Hypertension	153 (68.6%)	67 (63.2%)	86 (73.5%)
	Dyslipidemia	168 (75.3%)	82 (77.4%)	86 (73.5%)
	Other	62 (27.8%)	30 (28.3%)	32 (27.4%)
	HT + HCL	185 (83.0%)	88 (83.0%)	97 (82.9%)
	No	38 (17.0%)	18 (17.0%)	20 (17.1%)
Smoking (*n*, %)	No	141 (63.2%)	65 (61.3%)	76 (65.0%)
	Former	45 (20.2%)	21 (19.8%)	24 (20.5%)
	Yes/Secondhand smoker	37 (16.6%)	20 (18.9%)	17 (14.5%)
T2DM history (*n*, %)	≤15 years	96 (61.1%)	53 (70.7%)	43 (52.4%)
	>15 years	61 (38.9%)	22 (29.3%)	39 (47.6%)
Family history (PAD/Heart disease/Cerebrovascular disease) (*n*, %)	No	162 (72.6%)	81 (76.4%)	81 (69.2%)
	Yes	61 (27.4%)	25 (23.6%)	36 (30.8%)
Body composition, mean ± SD	Weight (kg)	66.9 ± 16.3	65.7 ± 14.1	67.9 ± 18.1
	Height (cm)	157.7 ± 8.8	158.5 ± 9.0	156.9 ± 8.6
	Waist circumference (cm)	91.9 ± 13.2	90.1 ± 11.8	93.5 ± 14.3
Physical activity level (*n*, %)	Low PA	117 (52.5%)	58 (54.7%)	59 (50.4%)
Activity conclusion (*n*, %)	Moderate PA	74 (33.2%)	35 (33.0%)	39 (33.3%)
	High PA	32 (14.3%)	13 (12.3%)	19 (16.2%)
Movement behavior (*n*, %)	Active	119 (53.4%)	55 (51.9%)	64 (54.7%)
	Sedentary	104 (46.6%)	51 (48.1%)	53 (45.3%)
ISWD, mean ± SD	-	235.8 ± 86.1	250.8 ± 88.9	222.1 ± 81.4

Note: cm; centimeters, HCL; hyperlipidemia, HT; hypertension, ISWD(m); the Incremental Shuttle Walk Distance, kg; kilograms, m; meters, *n*; number of participants, PA; physical activity, PAD; peripheral arterial disease, T2DM; type 2 diabetes mellitus, y; years.

**Table 2:** Receiver operating characteristic analysis of ISWD.

Parameters	Values
AUC (95% CI)	0.6 (0.54–0.67)
Optimal cut-point	215
Sensitivity (%)	62.6
Specificity (%)	58.3

Note: AUC, area under the curve; CI, confidence interval; ISWD, incremental shuttle walk distance.

**Table 3:** Factors associated with Peripheral Arterial Disease (PAD) (*n* = 223).

Factors	Coefficient (β)	OR	95% CI	*p*-Value
Age	0.024	1.02	0.99–1.06	0.192
Sex	−0.327	0.72	0.36–1.43	0.351
Diabetes Mellitus	1.144	3.14	0.24–41.04	0.383
Hypertension	0.605	1.83	0.90–3.71	0.093
Hyperlipidemia	−0.571	0.57	0.26–1.24	0.155
Smoking (Current)	0.072	1.08	0.47–2.48	0.865
Smoking (Former)	−0.335	0.72	0.33–1.54	0.394
Family history	0.307	1.36	0.72–2.58	0.347
Activity conclusion level 1 (high)	−0.410	0.66	0.16–2.74	0.570
Activity conclusion level 2 (moderate)	−0.291	0.75	0.31–1.82	0.521
Movement behavior	−0.034	0.97	0.27–3.41	0.958
ISWD ≤ 215 m	0.675	1.96	1.10–3.50	0.022 *

Note: Multivariable logistic regression analysis, * *p* < 0.05.

**Table 4:** Receiver operating characteristic analysis and optimal cut-off value for the risk prediction model.

Parameters	Values
AUC (95% CI)	0.67 (0.60–0.74)
Optimal cut-point	0.57
Sensitivity (%)	53.8
Specificity (%)	76.4
Youden’s index	0.30

Note: AUC, area under the curve; CI, confidence interval.

## Data Availability

The data presented in this study are available on request from the corresponding author. The data are not publicly available due to privacy and ethical restrictions.

## Abbreviations

The following abbreviations are used in this manuscript:

PAD	Peripheral arterial disease
T2DM	Type 2 diabetes mellitus
ISWT	Incremental shuttle walk test
ISWD	Incremental shuttle walk distance
ABI	Ankle–brachial index
TBI	Toe–brachial index
ROC	Receiver operating characteristic
AUC	Area under the curve
EPV	Events per variable

## References

[1] Criqui M.H., Aboyans V. (2015). Epidemiology of Peripheral Artery Disease. Circ. Res..

[2] McDermott M.M. (2015). Lower Extremity Manifestations of Peripheral Artery Disease: The Pathophysiologic and Functional Implications of Leg Ischemia. Circ. Res..

[3] Song P., Rudan D., Zhu Y., Fowkes F.J.I., Rahimi K., Fowkes F.G.R., Rudan I. (2019). Global, Regional, and National Prevalence of Peripheral Artery Disease in 2015. Lancet Glob. Health.

[4] Fowkes F.G.R., Rudan D., Rudan I., Aboyans V., Denenberg J.O., McDermott M.M., Norman P.E., Sampson U.K.A., Williams L.J., Mensah G.A. (2013). Comparison of Global Estimates of Prevalence and Risk Factors for Peripheral Artery Disease in 2000 and 2010: A Systematic Review and Analysis. Lancet.

[5] American Diabetes Association (2023). Standards of Medical Care in Diabetes—2023. Diabetes Care.

[6] Huang D., Refaat M., Mohammedi K., Jayyousi A., Al Suwaidi J., Abi Khalil C. (2017). Macrovascular Complications in Patients with Diabetes and Prediabetes. BioMed Res. Int..

[7] Tran B. (2021). Assessment and Management of Peripheral Arterial Disease: What Every Cardiologist Should Know. Heart.

[8] Høyer C., Sandermann J., Petersen L.J. (2013). Randomised Diagnostic Accuracy Study of a Fully Automated Portable Device for Diagnosing Peripheral Arterial Disease by Measuring the Toe-Brachial Index. Eur. J. Vasc. Endovasc. Surg..

[9] Aboyans V., Ricco J.B., Bartelink M.L., Björck M., Brodmann M., Cohnert T., Collet J.P., Czerny M., De Carlo M., Debus S. (2018). 2017 ESC Guidelines on the Diagnosis and Treatment of Peripheral Arterial Diseases, in collaboration with the European Society for Vascular Surgery (ESVS): Document covering atherosclerotic disease of extracranial carotid and vertebral, mesenteric, renal, upper and lower extremity arteriesEndorsed by: The European Stroke Organization (ESO)The Task Force for the Diagnosis and Treatment of Peripheral Arterial Diseases of the European Society of Cardiology (ESC) and of the European Society for Vascular Surgery (ESVS). Eur. Heart J..

[10] Norgren L., Hiatt W.R., Dormandy J.A., Nehler M.R., Harris K.A., Fowkes F.G.R. (2007). Inter-Society Consensus for the Management of Peripheral Arterial Disease (TASC II). J. Vasc. Surg..

[11] Hiatt W.R., Armstrong E.J., Larson C.J., Brass E.P. (2015). Pathogenesis of the Limb Manifestations and Exercise Limitations in Peripheral Artery Disease. Circ. Res..

[12] McDermott M.M., Guralnik J.M., Ferrucci L., Tian L., Pearce W.H., Hoff F., Liu K., Liao Y., Criqui M.H. (2007). Physical Activity, Walking Exercise, and Calf Skeletal Muscle Characteristics in Patients with Peripheral Arterial Disease. J. Vasc. Surg..

[13] Singh S.J., Morgan M.D.L., Scott S., Walters D., Hardman A.E. (1992). Development of a Shuttle Walking Test of Disability in Patients with Chronic Airways Obstruction. Thorax.

[14] ATS Committee on Proficiency Standards for Clinical Pulmonary Function Laboratories (2002). ATS statement: Guidelines for the Six-Minute Walk Test. Am. J. Respir. Crit. Care Med..

[15] Nantakool S., Chuatrakoon B., Sittichoke C., Konghakote S., Rerkasem K., Buranapin S., Kanlayanee S., Pothaya N., Kidarn J. (2024). A Comparison of Walking Performance Between Individuals with and without Asymptomatic Peripheral Artery Disease using the Six-Minute Walk Test and the Incremental Shuttle Walk Test. Sci. Prog..

[16] Holland A.E., Spruit M.A., Singh S.J. (2015). How to Carry Out a Field Walking Test in Chronic Respiratory Disease. Breathe.

[17] Banarjee C., Choudhury R., Park J.H., Xie R., Fukuda D., Stout J., Thiamwong L. (2024). Common Physical Performance Tests for Evaluating Health in Older Adults: Cross-Sectional Study. Interact. J. Med. Res..

[18] Hamburg N.M., Creager M.A. (2017). Pathophysiology of Intermittent Claudication in Peripheral Artery Disease. Circ. J..

[19] Hamburg N.M., Balady G.J. (2011). Exercise Rehabilitation in Peripheral Artery Disease: Functional Impact and Mechanisms of Benefits. Circulation.

[20] McDermott M.M. (2018). Medical Management of Functional Impairment in Peripheral Artery Disease: A Review. Prog. Cardiovasc. Dis..

[21] Purnamasari D., Tetrasiwi E.N., Kartiko G.J., Astrella C., Husam K., Laksmi P.W. (2022). Sarcopenia and Chronic Complications of Type 2 Diabetes Mellitus. Rev. Diabet. Stud..

[22] Arya S., Khakharia A., Rothenberg K.A., Johnson T.M., Sawyer P., Kennedy R.E., Brown C.J., Bowling C.B. (2020). Association of Peripheral Artery Disease with Life-Space Mobility Restriction and Mortality in Community-Dwelling Older Adults. J. Vasc. Surg..

[23] Roşu C.D., Bratu M.L., Stoicescu E.R., Iacob R., Hațegan O.A., Ghenciu L.A., Bolintineanu S.L. (2024). Cardiovascular Risk Factors as Independent Predictors of Diabetic Retinopathy in Type II Diabetes Mellitus: The Development of a Predictive Model. Medicina.

[24] Esmaeili P., Haybatollahi S.M., Roshanravan N., Ghaffari S., Alamdari N.M., Mousavi S., Asghari-Jafarabadi M. (2025). Cardio-Metabolic Risk Among Healthcare Providers: A Latent Profile Study. J. Cardiovasc. Thorac. Res..

[25] Treat-Jacobson D., McDermott M.M., Bronas U.G., Campia U., Collins T.C., Criqui M.H., Gardner A.W., Hiatt W.R., Regensteiner J.G., Rich K. (2019). Optimal Exercise Programs for Patients with Peripheral Artery Disease: A Scientific Statement From the American Heart Association. Circulation.

[26] Soyoye D.O., Abiodun O.O., Ikem R.T., Kolawole B.A., Akintomide A.O. (2021). Diabetes and Peripheral Artery Disease: A Review. World J. Diabetes.

[27] Rosana M., Yunir E., Saragih N., Rusdi L., Purnamasari D., Edi Tarigan T.J., Tahapary D.L., Soewondo P. (2025). Risk Factors for Peripheral Arterial Disease in Type 2 Diabetes Mellitus Patients: A Systematic Review and Meta-Analysis. Diabetes Res. Clin. Pract..

[28] Kim M., Kim Y., Ryu G.W., Choi M. (2021). Functional Status and Health-Related Quality of Life in Patients with Peripheral Artery Disease: A Cross-Sectional Study. Int. J. Environ. Res. Public Health.

[29] Dunn J., Runge R., Snyder M. (2018). Wearables and the Medical Revolution. Per. Med..

[30] Ribeiro R., Martins R., Pereira H., Crista V., Souza J., Almeida R., Martinho D., Conceição L., Freitas A., Marreiros G. (2026). A Systematic Review on Wearable-Enabled Remote Health Monitoring. Digit. Health.

